# Short-Term Epigenetic Responses of *Pinus brutia* to Fire Stress: Insights from a Prescribed Burning in Greece

**DOI:** 10.3390/genes17030309

**Published:** 2026-03-05

**Authors:** Evangelia V. Avramidou, Evangelia Korakaki, Nikolaos Oikonomakis, Miltiadis Athanasiou

**Affiliations:** 1 Institute of Mediterranean Forest Ecosystems, ELGO-DIMITRA, Terma Alkmanos, Ilisia, 11528 Athens, Greece; ekorakaki@elgo.gr; 2 School of Forestry and Natural Environment, Aristotle University of Thessaloniki, 54124 Thessaloniki, Greece; noikonomak@for.auth.gr

**Keywords:** methylation, prescribed burning, plant water potential, Fireline Intensity, mediterranean ecosystems

## Abstract

**Background/Objectives**: Fire is a dominant ecological force in Mediterranean ecosystems, shaping the adaptive traits of forest species such as *Pinus brutia*. Prescribed burning (also called controlled burning) is the intentional, carefully planned use of fire under specific environmental conditions to manage vegetation and reduce wildfire risk. While morphological and physiological fire adaptations are well-documented, emerging evidence highlights the role of epigenetic mechanisms—such as DNA methylation and histone modifications—in mediating stress responses. **Methods**: This study investigates genome-wide epigenetic changes in *P. brutia* following a prescribed burning experiment on Chios Island, Greece. Using methylation-sensitive amplified polymorphism (MSAP) analysis, we compared temporal shifts on epigenetic profiles before and after fire exposure extracting DNA from the same trees. **Results**: A significant increase in polymorphic epiloci, epigenetic diversity indices, and private epigenetic bands after prescribed burning was revealed, suggesting a stress-induced reprogramming of the epigenome. Concurrent measurements of midday needle water potential indicated an exploratory association between water stress and epigenetic shifts. Furthermore, Fireline Intensity (FI) correlated with epigenetic diversity index signaling an immediate response of the tree. **Conclusions**: These findings support the hypothesis that fire stress induces epigenetic responses in *P. brutia*, potentially enhancing resilience to future environmental challenges. Further research is required to address the level of heritability of these epigenetic changes in next generation and connect these indexes with adaptation and sustainability of forest ecosystems.

## 1. Introduction

Fire and heat stress are major environmental pressures that shape the evolution and adaptability of forest tree species, including pine trees [1]. While the physiological and morphological adaptations of pines to fire—such as serotiny and thick bark—are well-documented, emerging evidence suggests that epigenetic mechanisms may also play a critical role in mediating responses to thermal stress. Epigenetic modifications, such as DNA methylation, histone modifications, and non-coding RNAs, can regulate gene expression without altering the underlying DNA sequence, allowing trees to rapidly adjust to changing environments [2,3]. In pine species, exposure to fire or heat may trigger such modifications, leading to “stress memory” that can persist over time and potentially across generations [4]. For example, in *Pinus pinaster* and *Pinus halepensis*, fire-related selection has been associated with adaptive traits like serotiny, which may be partly under epigenetic control [5,6]. Understanding how fire-induced epigenetic reprogramming occurs in pines is essential not only for evolutionary biology but also for forest management and conservation in the context of climate change and worsening fire regimes. Another parameter that should be considered is the change in phenotype in burned and unburned plants that is happening according to [7] that may affect plant- animal interactions also.

*Pinus brutia* (commonly known as Turkish pine) is one of the most ecologically and economically important conifer species in Greece. It typically grows at low-to-mid elevations (0–800 m) in areas with dry, hot summers and mild, wet winters. It is naturally distributed across southern and eastern Greece, including many Aegean islands such as Crete, Rhodes, Samos, Chios, and Lesvos, as well as coastal and low-elevation areas of the Peloponnese, Sterea Ellada, Thessaly, and parts of Macedonia [8]. The species typically occupies elevations from sea level up to 800 m, thriving on calcareous and siliceous substrates under the hot, dry summers and mild, wet winters characteristic of the Mediterranean climate. Ecologically, *P. brutia* is highly adapted to fire-prone environments. It exhibits several traits that confer resilience to wildfire, including thick bark, rapid growth, and an ability to regenerate post-fire via both seed germination and resprouting [9]. These fire-adaptive characteristics make it a keystone species in many Mediterranean pine ecosystems, where it supports biodiversity, prevents soil erosion, and contributes to landscape stability [8]. Its forests are also valuable as habitat for numerous plant and animal species, maintaining important ecological processes in regions where degradation and desertification risks are high. From an economic perspective, *P. brutia* has long been used for timber and firewood, but it is especially valued for resin production, a traditional industry in many Greek regions [10]. Its wood is moderately durable and used in construction and furniture, while the species is also heavily planted in reforestation projects due to its fast growth and tolerance to poor soils.

Two studies in *Pinus brutia* forests in Marmaris (Turkey) and Thasos (Greece) examined how the species regenerates after fire. Burned sites of different ages and conditions were tracked to measure seedling emergence, survival, growth, and reproduction. Both areas showed a single flush of seedlings in the first year, followed by high mortality and stable sapling numbers within 2–5 years. Trees grew about 17 cm per year, especially on fertile, north-facing slopes. Some began reproducing by age four, and by year nine, up to 15% produced cones with viable seeds. These results highlight *P. brutia’s* strong resilience and the ecological importance of fire-driven regeneration in Mediterranean forests [11,12]. In the context of climate change, *P. brutia* is of growing interest because of its drought and fire resilience. However, increasing fire frequency, prolonged droughts, and the spread of pests and pathogens pose significant threats to their long-term survival. Therefore, conservation strategies should focus on identifying and protecting genetically and epigenetically resilient populations, particularly in regions where the species reaches its ecological limits (e.g., southern Aegean islands. Studying intraspecific variation in stress responses—including through epigenetic mechanisms—could be key for ensuring the adaptability and sustainability of pine forests in future Mediterranean landscapes.

Prescribed burning (PB) is a fuel-management method that supports biodiversity conservation [13,14], enhances forest ecosystem resilience, and contributes to reducing wildfire hazard and risk. This study aims to bridge the gap by integrating epigenetic temporal responses of *P. brutia* into fire stress. Specifically, the following key research questions are addressed: (1) What epigenetic changes occur in the trees before and after the fire exposure? (2) What is the correlation with water stress dynamics? (3) Can epigenetic status provide insights for future conservation actions, or can it be used for more resilient trees related to fire intensity?

## 2. Materials and Methods

### 2.1. Study Site

**Figure 1:**
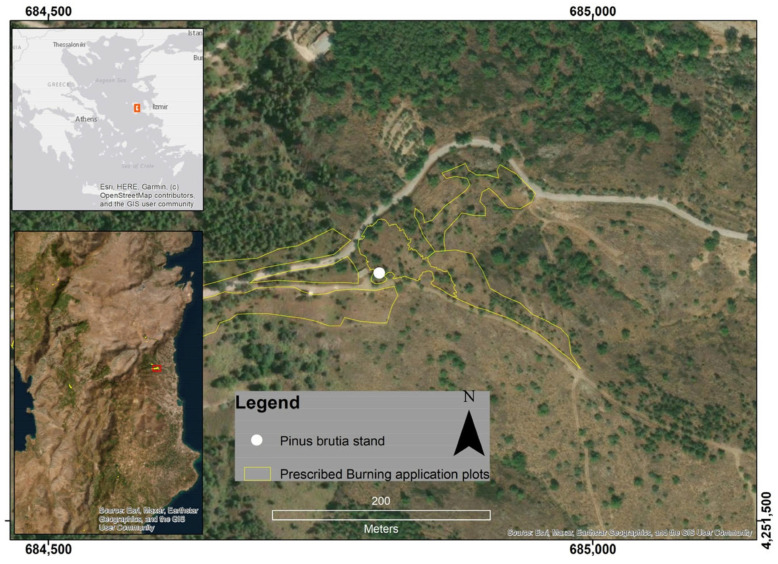
The Greek Grid System (EGSA87) was used to project the study area on the island of Chios, Greece. The red squares in the two locator maps show the location and size of the study area. ArcGIS version 9.3 Geographic Information System software, developed by ESRI, was used to create the map.

**Figure 2:**
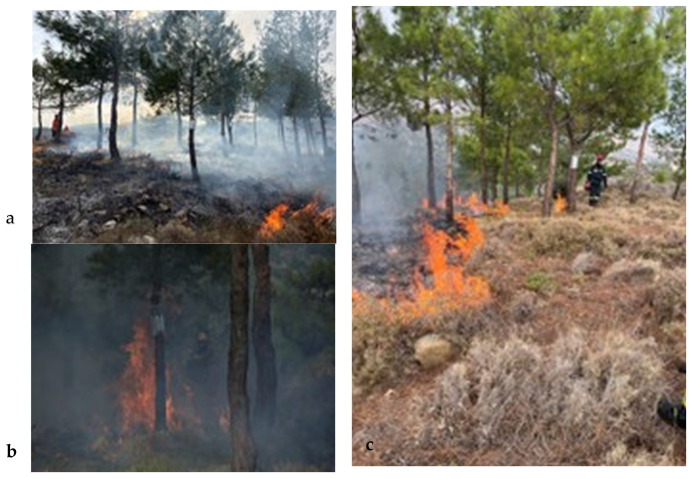
From right to left: (**a**) study site; (**b**) prescribed burning in progress; and (**c**) studied *P*. *brutia* trees.

In 2021, a pilot project for prescribed burning was launched on the island of Chios, Greece (Figure 1) for *P. brutia* ecosystem. A series of parameters were measured before, during and after the implementation of prescribed burning (Figure 2) [15]. These included meteorological conditions and Flame Length (FL, m). Using the recorded Flame Length values, we subsequently calculated Fireline Intensities (FI, kW·m^−1^) [16,17].

The measurement of Flame Length was taken in a relatively flat area following specific procedures [18,19,20,21,22,23]. The steps involved in the measurement process were as follows: the geographic coordinates of the observer’s positions were obtained using a GPS device. For ground-based photography, images of the fire perimeter at sequential locations were captured using a Canon EOS 70D DSLR camera. The horizontal azimuth for each photograph was recorded using a compass. The camera automatically embedded metadata in the digital image files, including the time each photo was captured. Flame Length was calculated using straightforward geometric techniques. Because each observation was stamped, it could be directly paired with the corresponding fine live or dead fuel-moisture measurements taken independently.

Fireline Intensity (FI) was then calculated from Flame Length using the appropriate method, depending on fuel type and observed Flame Length. Specifically, we used the following equations (also presented in Alexander and Cruz [24]).

(a) For fire behavior in *Sarcopoterium spinosum*-dominated shrublands, FI (kW m^−1^) was calculated using the equation developed by Fernandes et al. [24]:

$$FI=695.0\times {FL}^{2.21}$$

where FI is Fireline Intensity (kW m^−1^) and FL is Flame Length (m). The values of FL (m) ranged from 0.2 to 3.1 and for FI 12–7605 (kW m^−1^).

For fire behavior in Mediterranean pine forest litter and shrubs under, FI was calculated using the equation developed by Nelson and Adkins [25]:

$$FI=483.3\times {FL}^{2.03}$$

The values of FL ranged from 0.5 to 32.5 (m) and for FI 98–2755 (kW m^−1^).

Weather data were obtained through direct field measurements, while fuel characteristics were inferred using functions developed specifically for the fuel types. Fire spread was measured directly by the authors. Hence, the reliability of the methods and measurement techniques used to assess meteorological conditions, describe forest fuels and document fire rate of spread and behavior was the highest, according to [25].

### 2.2. DNA Sampling and Extraction

Needles from the same four trees were collected for DNA extraction before and immediately after the prescribed burning. For the purposes of this study, samples collected before and after the fire were treated as indicative populations. A commercial plant DNA extraction kit (Ma-cherey-Nagel, Düren, Germany) was used to extract genomic DNA. The concentration of DNA was measured using a UV spectrophotometer (Eppendorf BioPhotometer, Hamburg, Germany).

### 2.3. Methylation-Sensitive Amplification Polymorphism (MSAP) Procedure

The MSAP assay was carried out using the primers presented in Table 1 and EcoRI/HpaII or EcoRI/MspI restriction enzymes for double digestion. The whole procedure is described analytically in our previous publication [26]. Moreover, three samples were used as replicate analyses, employing the same DNA extractions for the MSAP analysis.

Allele size data from GeneMapper 4.0 (Applied Biosystems, Foster City, CA, USA) was converted into a binary format for the MSAP analysis. Then, Excel macros were used to mark allele sizes with 1 if present and 0 if not. Potential size homoplasy was minimized by only considering and analyzing further reproducible fragments between 150 and 500 base pairs; see [27] for more details. Moreover, the results of the comparison of banding patterns from EcoRI/HpaII and EcoRI/MspI reactions are presented in four possible fragment conditions according to [28] for MSAP analysis: (I) fragments present in both profiles (1/1), indicating an unmethylated state (u alleles); (II) fragments present only in the EcoRI/MspI profile (0/1), indicating hemi- or fully methylated CG sites (m alleles); (III) fragments present only in the EcoRI/HpaII profile (1/0), suggesting hemimethylated CHG sites (h alleles); and (IV) absence of fragments in both profiles (0/0), representing an uninformative state, which may be due to various methylation types or restriction site polymorphisms. To differentiate between unmethylated and methylated fragments and to evaluate the specific impact of methylation conditions II and III, the ‘Mixed-Scoring 2’ approach was employed [28].

### 2.4. Needle Water Potential

Needle water potentials (Ψ) were measured before and after the prescribed fire on pine trees grown under uniform environmental conditions. The same four individuals used for epigenetic sampling were included in these measurements. Four *P. brutia* individuals per natural population were sampled (twigs with needles). A portable pressure chamber (model PMS 1003, PMS Instruments, Corvallis, OR, USA) was used to conduct Ψ measurements between 10:00 and 13:00 [20].

### 2.5. Statistical Analysis

Data processing and statistical analyses were performed using the R software environment for statistical computing (version 4.3.1; R Core Team, Vienna, Austria, 2023). To assess the relationship between Fireline Intensity (kW/m) and the ecological indices (Iepi), we employed Pearson’s product–moment correlation coefficient (r) and ordinary least squares (OLS) linear regression. Given the constraints of the dataset size (n = 4), all regression analyses are presented as exploratory. Model performance was evaluated using the coefficient of determination (R^2^) and the standard error of the estimate. To account for the statistical uncertainty inherent in the small sample size, we calculated and visualized 95% confidence intervals for all regression slopes. Although the sample size (n = 4) limits the power of normality testing, Pearson’s correlation coefficient was selected to specifically evaluate the linearity of the relationship between Fireline Intensity and Iepi. Non-parametric rank methods were not utilized as they assess only monotonic ordering and do not quantify the linear proportionality required for potential intensity calibration. The distinction between pre-fire and post-fire states was evaluated using paired comparisons of the index values. All graphical visualizations were generated using the ggplot2 [29] and ggpubr [30] packages.

## 3. Results

Observed Fireline Intensity (FI) varied from 0.5 to 2 m, corresponding to Fireline Intensity of 118 to 3216 kW·m^−1^. These observations were made under mild weather conditions, with an air temperature of 16οC, 62% relative humidity, and a light breeze.

### 3.1. Epigenetics Results

Six MSAP selective primer combinations were used and produced 746 fragments for the two populations studied herein. The results for epigenetic parameters were different concerning loci before and after fire populations (Table 2). Mean percentage of polymorphism was 91.29% and 38.34% for after and before fire populations respectively. The mean unbiased Heterozygosity (uHe), the mean Epigenetic Shannon Index (Iepi), the number of private epigenetic bands (NPB) were all higher after the fire, indicating plastic responses of the tree handling the thermal stress that occurred. The genotyping error rate, estimated from replicate samples, was approximately 2–3%.

**Figure 3:**
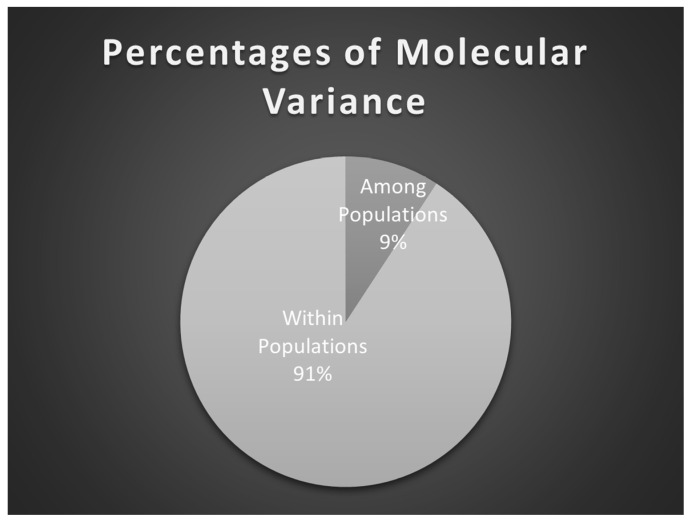
Percentage of molecular variance among and within populations.

Analysis of molecular variance portioned 91% of the epigenetic variation within populations and 9% only among populations for all epiloci (Figure 3).

**Figure 4:**
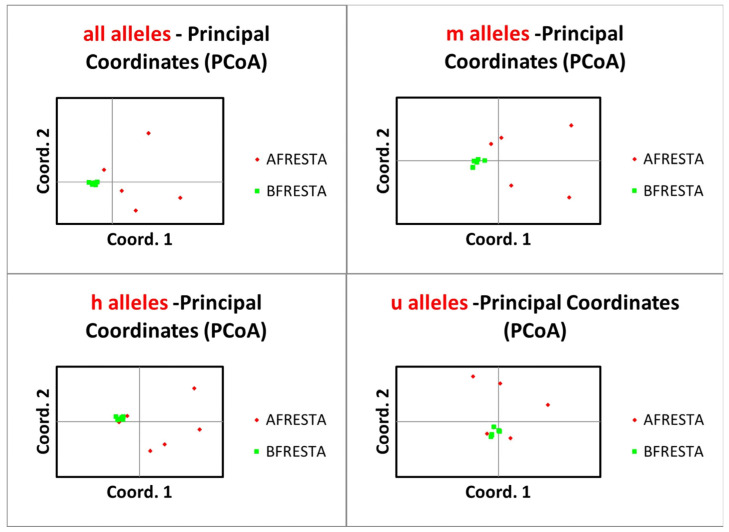
Principal coordinate analysis of epigenetic (MSAPs), for all alleles and three distinct methylation types: m, u, and h alleles for before and after fire populations.

Principal coordinate analysis for (1) all alleles, (2) m alleles, (3) h alleles, and (4) u-alleles for before fire and after fire loci populations, explaining 39.40%; 34.83%; 38.61% and 39.37%, respectively (Figure 4).

### 3.2. Correlation of Needle Water Potential with Epigenetic Shannon Index (Iepi)

**Figure 5:**
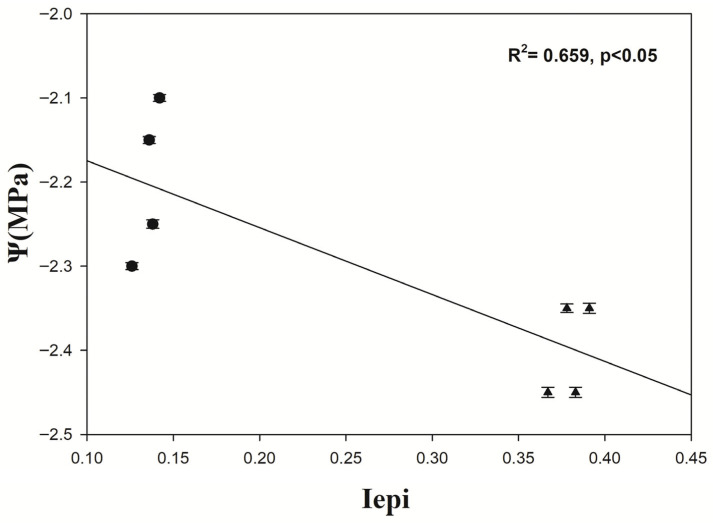
Correlation of needle water potential with Iepi, before (circles) and after prescribed fire (triangles).

There is an indicative strong correlation between needle water potential (Ψ) and Iepi (R^2^ = 0.659, *p* < 0.05). In addition, a clear separation is observed between pre-fire and post-fire samples: post-fire samples (triangles) exhibit the highest Iepi values, whereas pre-fire samples (circles) show the lowest values (Figure 5).

**Figure 6:**
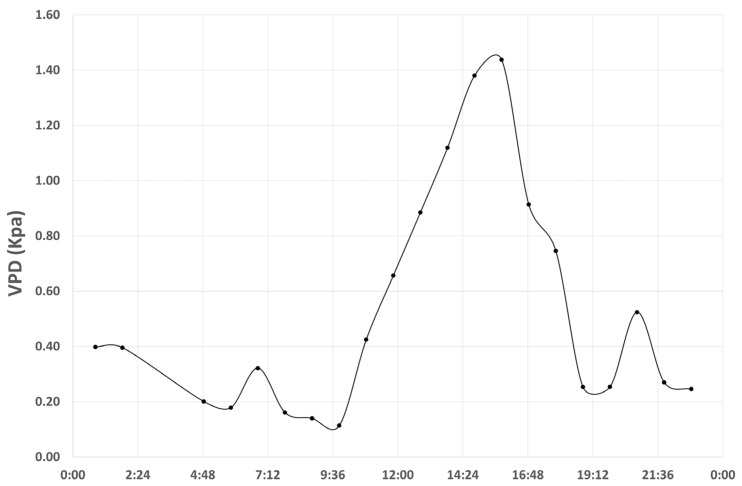
Diurnal variation in vapor pressure deficit (VPD) on 7 December 2022.

The diurnal course of air temperature and relative humidity on 7 December 2022 explains the observed variation in vapor pressure deficit (VPD, Figure 6). As air temperature increased from late morning and reached its maximum around 14:00, relative humidity declined correspondingly. This combination of higher temperatures and reduced atmospheric moisture resulted in a pronounced increase in VPD during the late afternoon. Subsequently, the decrease in temperature and concurrent rise in relative humidity led to a progressive reduction in VPD.

**Figure 7:**
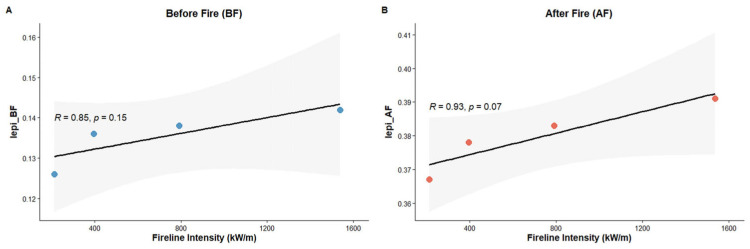
Correlation of Fireline Intensity FI with Iepi, before (**A**) and after prescribed burning (**B**).

We also used correlation to assess the relationship between Iepi before prescribed fire and Fireline Intensity values (kW·m^−1^) and after fire (Table 3, Figure 7). Given the limited sample size (n = 4), the relationship between Fireline Intensity (FI) and the Iepi index is characterized as exploratory. While a strong positive linear trend was observed post-fire (R^2^ = 0.865), the 95% confidence intervals for the slope cross zero (Table 3), reflecting the statistical uncertainty associated with the small dataset.

Notably, the analysis reveals a strong pre/post separation that dominates the signal. The mean index value increased by Δ = 0.244 (an ≈180% increase) immediately following the fire event. This suggests that the binary occurrence of fire (burned vs. unburned) acts as the primary driver of the index shift, while the specific intensity of the fire front (Ψ) plays a secondary, modulating role. The residual variance in the linear model (1 − R^2^ ≈ 0.135) may be attributed to confounding local factors such as soil moisture heterogeneity or variations in residence time that were not explicitly captured by the FI metric.

## 4. Discussion

Climate change poses a significant threat to forest health, making adaptation crucial for maintaining resilient forests. Trees, being sessile, can adjust their phenotypes to cope with environmental changes. While natural selection can drive genetic adaptations, this process is slow, especially in long-lived species. A faster alternative mechanism is phenotypic plasticity, often regulated by epigenetic mechanisms [7], which allows quicker adjustments within and across generations [31]. Although fires and temperature rising significantly affect tree mortality, few studies concerning the epigenetic integrity of the forest trees exist [32,33]. Extreme heat events, such as heat waves, are rising in frequency and can cause tree mortality [34]. Plant responses to heat are also influenced by epigenetics [35,36]. Lamelas et al. (2020) [36] studied Monterey pine (*Pinus radiata*) under heat stress, tracking protein expression and DNA methylation in needles. They found proteins linked to chromatin reorganization, suggesting epigenetic involvement in thermopriming, supported by heat stress markers like heat shock proteins. Another example of heat stress involves the cork oak tree. Cork oak (*Quercus suber*), a native tree to the western Mediterranean, showed high heat and drought tolerance [37,38] when subjected 8-month-old trees to a temperature ramp from 25 °C to 55 °C over 12 days. They found a 0.5% increase in genome-wide methylation and decreased histone acetylation, suggesting epigenetic mechanisms contribute to heat tolerance.

Herein we studied before and after fire *P. brutia* populations to clarify if the temperature stress that trees face under a prescribed burning experiment modifies their temporal epigenetic integrity. We studied changes that happened in the primary face in the needles of the same trees before and after prescribed burning. Our results agree with previous in-depth experiments mentioned above for *P. radiata* [36] and *Q. suber* [38] and pinpoint a increase in DNA methylated epiloci in the whole genome. Our results revealed higher values of epigenetic diversity indices in post-fire samples compared to pre-fire samples of the same trees. Importantly, these changes should be interpreted as reflecting increased variability at methylation-sensitive CCGG loci, rather than a direct or global increase in overall genome-wide methylation levels. Given the locus-specific nature of the MSAP approach, conclusions regarding total methylation levels across the genome cannot be drawn. These first results can primarily be a base point for further exploitation of genes that accumulate temperature rising. Furthermore, if we consider the fact that *P. brutia* has traits that enhance wildfire resilience, such as thick bark, rapid growth, and the ability to regenerate through both seed germination and resprouting [9], we may connect its temperature resilience with these epigenetics temporal shifts.

In the context of prescribed burning (PB) numerous studies showed that biodiversity is enhanced [39,40] and simultaneously, trees can strongly be adapted to the fire stress through epigenetics modifications. In this study, the correlation between midday water potential (Ψ) and the epigenetic diversity index (Iepi) reveals a clear separation between pre-fire and post-fire samples. Post-fire samples are characterized by higher Iepi values, whereas pre-fire samples exhibit lower values. While this pattern suggests an association between fire exposure and increased epigenetic variability, it must be interpreted with caution. The observed relationship likely reflects the strong temporal separation between sampling periods rather than a direct physiological linkage between Ψ and epigenetic diversity. Moreover, midday Ψ is highly sensitive to short-term atmospheric conditions, such as vapor pressure deficit, which may have contributed to the observed differences. Even relatively mild stress conditions could therefore trigger or amplify epigenetic responses detectable by MSAP markers.

Regarding the relationship between Fireline Intensity (FI) and Iepi, correlation coefficients were large in magnitude but not statistically significant due to the very small sample size. These results are therefore considered exploratory and hypothesis-generating. While they are consistent with the idea that trees may respond to increasing fire intensity through epigenetic mechanisms, additional data is required to robustly evaluate the strength and significance of this association.

Furthermore, we should mention that the correlation analyses presented in this study are limited by small sample sizes, which reduce statistical power and preclude more robust inferential approaches, such as stratified or resampling-based analyses. In addition, the strong separation between pre- and post-fire sampling periods may confound the observed relationships, particularly for midday Ψ, which is highly influenced by environmental conditions such as vapor pressure deficit. Consequently, the reported correlations should be interpreted as exploratory and serve primarily to guide future studies with increased replication and finer temporal resolution.

The answer to question whereas the epigenetic status of tree populations offers valuable insights that can significantly inform conservation and forest management strategies we can connect identify epigenetic biomarkers. By characterizing the epigenetic landscape across different regions, researchers can pinpoint biomarkers that are specific to environmental stressors [41]. These biomarkers enable rapid assessments of a tree population’s stress levels or overall health—providing information far more quickly than traditional measures like ecophysiological traits or demographic rates.

Epigenetic information may also help identify tree populations that appear pre-adapted to environmental conditions. These populations could be prioritized as source material for reforestation or restoration programs, thereby increasing the likelihood that planted trees will successfully establish and persist under challenging conditions [26,32]. Furthermore, the possibility of transgenerational inheritance [42] of beneficial epigenetic modifications presents a groundbreaking opportunity. If such inheritance is confirmed in trees—as it has been in other plant species—it could represent a rapid, natural mechanism for stress adaptation across generations, accelerating evolutionary responses in forest ecosystems. However, the extent and ecological relevance of such inheritance in long-lived tree species remain largely unresolved and require further investigation.

Beyond its diagnostic value, epigenetics has been proposed as a potential tool for proactively enhancing tree resilience, including resilience to fire-related stress. Although fire is not always treated as a distinct stress factor, it induces intense heat and can increase susceptibility to subsequent biotic and abiotic challenges. Experimental work on thermal and defense priming suggests that prior exposure to stress can induce epigenetic changes that influence subsequent responses [43,44]. Thermal priming, for example, involves exposing embryos or somatic tissues to high temperatures—a process known as thermopriming—which has been shown to create long-term epigenetic memory. In species like *Pinus radiata*, this memory is linked to proteins involved in chromatin reorganization, suggesting that heat exposure can induce lasting epigenetic changes. This technique opens the door to developing stress memory in trees as a method of sustaining forest health under climate stress [36]. While such findings highlight the potential of epigenetic mechanisms to contribute to stress tolerance, their long-term stability and applicability in forestry remain to be fully demonstrated. Integrating epigenetic approaches into experimental research and applied forest management may ultimately support the development of more resilient forests, but this will require rigorous validation across species, environments, and time scale [32,43].

Finally, to effectively incorporate epigenetics into forestry and conservation practices, several areas require future investigation. Future research is needed in deciphering epigenetic mechanisms and memory duration; this is a crucial area for future experiments that involve gaining a better understanding of the intricate epigenetic mechanisms that contribute to heat stress memory in forest trees, as these mechanisms are still not fully understood. For example the extent to which gymnosperms possess a heat stress memory like angiosperms remains largely unexplored although that some initial experiments in maritime pine (*Pinus pinaster*) to heat shock before embryogenesis resulted in plants that were more resilient to heat stress and retained that memory for at least 2 years [45,46]. Moreover, it is vital to quantify the rates and shapes of epigenetic shifts (e.g., methylation changes) over a single plant generation and track long-term trends of epigenetic flux (over decades/centuries) to see how these changes are inherited [32].

## 5. Conclusions

In this study, we found an indication that exposure to fire stress for *P. brutia* triggered immediate epigenetic temporal changes, as evidenced by a sharp increase in the mean percentage of epigenetic polymorphism from 38.34% before the fire to 91.29% immediately after in the same trees studied. This molecular response included a rise in polymorphic epiloci, epigenetic diversity indices, and private epigenetic bands, suggesting a rapid plastic adjustment to thermal stress. Research indicated a correlation between midday needle water potential and the Epigenetic Shannon Index, which points to a link between water stress dynamics and these epigenetic shifts. Furthermore, preliminary data indicate a positive association between the ecological response and Fireline Intensity (FI). Although the sample size limits definitive conclusions, the results suggest that the index may become increasingly sensitive to fire intensity after a burning event has occurred, warranting further investigation with larger datasets. These modifications, particularly DNA methylation, facilitate the creation of a “stress response” that may persist or not across generations, allowing long-lived trees to adapt to changing environments without altering their underlying DNA sequence. Future research in identifying epigenetic biomarkers and utilizing techniques like thermopriming can significantly assist conservation strategies by identifying pre-adapted populations and enhancing forest resilience against future climate-driven fire regimes.

## Figures and Tables

**Table 1:** EcoRI/HpaII-MspI adapters, pre-selective and selective primers used for the MSAP analysis.

Primer Name	5′ to 3′ Sequence
EcoRI adapter	CTCGTAGACTGCGTACC AATTGGTACGCAGTC
HpaII/MspI adapter	GACGATGAGTCTCGAT CGATCGAGACTCAT
Pre-selective EcoRI primer	GACTGCGTACCAATTC-A
Pre-selective HpaII/MspI primer	ATGAGTCTCGATCGG-A
Selective EcoRI primers	GACTGCGTACCAATTC + ATG (FAM) GACTGCGTACCAATTC + ACT (HEX) GACTGCGTACCAATTC + AAC (ROX) GACTGCGTACCAATTC + AAG (TAMRA)
Selective HpaII/MspI primer	ATGAGTCTCGATCGGATC ATGAGTCTCGATCGGACT ATGAGTCTCGATCGGAAT

**Table 2:** Populations after fire (AFRESTA) and before fire (BFRESTA); Pepi: percentage of polymorphic subepiloci; Na: number of alleles; Ne: number of effective alleles; Iepi: Shannon’s information index based on epiloci; uHe: unbiased epigenetic diversity; N.B.: number of epigenetic bands; N.P.B.: number of private epigenetic bands.

Pop		Pepi	Na	Ne	Iepi	uHe	NB	NPB
AFRESTA	Mean	91.29	1.827	1.350	0.383	0.258	681	452
	SE		0.021	0.009	0.006	0.005		
BFRESTA	Mean	38.34	0.777	1.120	0.143	0.093	286	64
	SE		0.036	0.007	0.007	0.005		

**Table 3:** Exploratory regression analysis of Fireline Intensity (FI) effects on Iepi indices (n = 4).

▪ Dependent Variable	Slope (*β*)	Std. Error (SE)	95% Confidence Interval for *β*	Coefficient of Determination (R^2^)	*p*-Value	Mean Value
▪ **Pre-Fire** **(I_epi_BF_)**	9.81 × 10^−6^	4.37 × 10^−6^	[−8.99 × 10^−6^, 2.86 × 10^−5^]	0.716	0.154	0.136
▪ **Post-Fire** **(I_epi_AF_)**	1.59 × 10^−5^	4.44 × 10^−6^	[−3.21 × 10^−6^, 3.5 × 10^−5^]	0.865	0.070	0.380
▪ **Difference (Δ)**	-	-	-	-		+0.244

## Data Availability

Dataset available on request from the authors.
